# Who Will Score? A Machine Learning Approach to Supporting Football Team Building and Transfers

**DOI:** 10.3390/e23010090

**Published:** 2021-01-10

**Authors:** Bartosz Ćwiklinski, Agata Giełczyk, Michał Choraś

**Affiliations:** Faculty of Telecommunications, Computer Science and Electrical Engineering, UTP University of Science and Technology, 85-796 Bydgoszcz, Poland; bartosz.cwiklinski@utp.edu.pl (B.Ć.); chorasm@utp.edu.pl (M.C.)

**Keywords:** machine learning, big data, football support, sports analytics

## Abstract

Background: the machine learning (ML) techniques have been implemented in numerous applications, including health-care, security, entertainment, and sports. In this article, we present how the ML can be used for building a professional football team and planning player transfers. Methods: in this research, we defined numerous parameters for player assessment, and three definitions of a successful transfer. We used the Random Forest, Naive Bayes, and AdaBoost algorithms in order to predict the player transfer success. We used realistic, publicly available data in order to train and test the classifiers. Results: in the article, we present numerous experiments; they differ in the weights of parameters, the successful transfer definitions, and other factors. We report promising results (accuracy = 0.82, precision = 0.84, recall = 0.82, and F1-score = 0.83). Conclusion: the presented research proves that machine learning can be helpful in professional football team building. The proposed algorithm will be developed in the future and it may be implemented as a professional tool for football talent scouts.

## 1. Introduction

Sports have been one of the most popular kind of entertainment for ages. Practising and watching sport is exciting, healthy, unpredictable, and makes us feel alive. However, sports have recently become very lucrative business. Sports leagues and teams turned into industries, earning billions of dollars from various sources: sponsorships, ticket revenues, transfers, stadium rentals, broadcasting deals, merchandise, and many more. The revenue of sports leagues is constantly growing, according to the online reports (e.g., by Athletic Panda https://apsportseditors.org/others/most-profitable-sports-leagues/). In 2019, the biggest one was generated in the US American football league, National Football League (NFL)-$13 Billion. Other sports can also be very profitable: in 2019, Major League Baseball (MLB) earned $10 Billion, National Basketball Association (NBA) $7.4 Billion, Indian Premier League (Cricket) $6.3 Billion, and, finally, the English Premier League made $5.3 Billion.

Because sports teams have become enterprises with millions of budget funds, they have to generate profits. Thus, they need to be managed carefully and reasonably. Player transfers is one of the tasks that managing a sports team encompasses. Hence, in the article, we provide a system that can support the football team building and transfers. It uses the machine learning techniques and it has been tested on the real-life data, which is publicly available online.

The popularity of machine learning and artificial intelligence (AI) has recently increased rapidly. Currently, they are widely implemented in many domains of our everyday life, including sports. AI provides the opportunity to simulate human-like thinking in computer systems. However, it also enables analysing large amounts of data, which may prove to be hardly executable for humans.

Sport is a perfect example of a field producing thousands bytes of data. A single football match (45 min. each half) can generate an enormous amount of data: goals, attempts, attempts on goal, corners, yellow and red cards, time on field of each player, substitutes, ball possession, and many others. However, is every piece of information valuable? Or, on the other hand, are there any other types of data that can be useful in terms of player evaluation, transfer, and, finally, the whole sport team building?

The major contribution of this paper is to present possible implementation of the machine learning techniques in order to predict a successful transfer of a professional football player. The presented solution uses realistic data and different definitions of transfer success. It is also remarkable that the algorithm pays attention to all aspects of a player: his technical, physical, and psychological state.

The remainder of the paper is structured, as follows: Section 2 presents the state of the art and some existing scouting tools. In Section 3, the proposed solution is described in detail. Section 4 contains and discusses the obtained results. Section 5 and Section 6 provide threats to validity and conclusions, respectively.

## 2. Related Work and Existing Solutions

At the very beginning, the term ’sports analytics’ needs to be defined. According to [1], this term can be understood as ’statistics in sports’, and it encompasses data collection and management, predictive modeling, and computational methods that are used to find valuable information for sport-related decision making. From the scientific perspective, it can be covered by the collection and analysis of past and current sports data (obtained from boxscores, videos, demographics, medical, and scouting reports). It can also be extremely important and informative for team staff, coaches, clubs owners, and every single player.

Traditionally, the results of matches were predicted using some mathematical and statistical models, and they were often verified by a domain expert, as mentioned in [2]. Currently, it is possible to predict the sport outcomes by means of machine learning and/or artificial intelligence. The authors of [3] presented the review of the current state-of-the-art in this area. They pointed out the main problem in comparing different methods and their results, which results from the fact that almost every paper uses a different dataset. They also emphasised the fact that surprises in sport happen on a daily basis and they are usually difficult to predict.

The possible approach for predicting the outcomes was presented in [4], where the NBA results are forecast. The authors used different classifiers: the Naive Bayes, Articial Neural Network, and decision trees. They used various sets of features that are related to basketball games, and, thus, they were able to discover the key features that provide better performance (accuracy and efficiency) of the prediction model. On the other hand, the football outcome predicting framework was presented in [5].

In [6], the validation step was investigated. The authors claimed that there are two possible validation methods for predicting the results of basketball matches, namely train & test and cross-validation. The presented results proved that the cross-validation provided better accuracy for the following classifiers: logistic regression, Naive Bayes, a decision tree, kNN (k-nearest neigbors), Random Forest, and LogiBoost.

Not only the basketball results are predicted using machine learning. In [7], the badminton scores are predicted, [8] concerns football football match scores, while [9] predicts the hockey results.

Even though the tweets’ content is mostly analysed in order to uncover the fake pieces of information (as presented in [10]), the authors of [11] based the results’ prediction on the tweets’ sentiment analysis.

Some of the researchers used machine learning in order to assess the risk or predict an injury, as an inline player’s injury can be a critical factor resulting in a victory or defeat. In [12], the injury risk was estimated for professional rugby league players. In the research, ANN (Artificial Neural Network) and RF (Random Forest) were used. The used dataset was dedicated to this research. It contained the data that were collected from 46 professional rugby league players throughout the 2015 NRL season, including some data collected by GPS (Global Positioning System). Similar approaches were presented in [13,14]. In these articles, the authors proposed their systems for predicting the possibility of the injury of the football players.

The authors in [15] were exclusively focused on football. They summed up the usage of machine learning in this area and highlighted that possessing even bigger and bigger amounts of data could bring about a revolution in football analytics. As potential fields for implementing ML, they indicated tactic improvements, discovering the factors that lead to goal scoring, the identification of the opposing team’s strengths and weaknesses before the match, and determining the areas that a team needs to improve in.

The article [16] presents the machine learning approach for creating a ranking of professional football players. The main aim of the research is to provide a data-driven framework that offers a role-aware evaluation of football players’ performance. The ranking estimation is also presented in the article [17], in which a generic algorithm was used in the voting part of the system.

Because, across countries and continents, football has drawn increasingly more attention, more bookmakers are offering football bets. The scientific approaches to beating the bookmakers were presented in [18,19].

It is also possible to combine machine learning with computer vision in the sport domain, as presented in [20]. The article presents the basketball players’ movement recognition and its classification as a shoot, a pass, a catch, or a dribble.

Machine learning can also be implemented in order to predict the future performance of athletes. This kind of analysis can be especially beneficial for the coaches of young, promising players, or the scouts looking for new sport stars. The possible implementations of machine learning in performance prediction were presented in [21]-in tennis, [22]-handball, and in [23]-archery.

However, the machine learning-based solutions could be useful not only for the professional athletes, but also for amateurs. The wearable devices have recently become increasingly popular. In [24], a possible implementation of the machine learning techniques in the wearable devices was presented. Thanks to this kind of solution, a person can constantly monitor their health. On the other hand, in [25], a ML-based framework for training plan generation in coaching was proposed. In the sport jargon, a person working on transfers and statistics is called a scout. There are plenty of professional tools supporting scouts in their duties. However, they technically do not provide any transfer suggestions (they can only present some statistics on the potential player), or they treat the transfer management as a black box. The first one is Scoutactic (https://scoutastic.com/en/). Among its functionalities, the following are enumerated: team management-dynamic resource planning, task assignment, ticket status, area match search, and scouting-activity analytics; match report analysis-extracting, displaying and filtering the relevant content from match reports; and, data generation-automated generation of the relevant performance data and development indicators while using the AI analysis of videos (training, test matches, and TV broadcasts). However, it does not support team building and transfers.

Wyscout is the other platform dedicated to scouts (https://wyscout.com/). It is the world’s leading provider of football performance data. It provides advanced statistics that can help coaches to analyse and prepare matches, give scouts powerful tools to identify the most promising profiles, and enable player agents to better understand their players’ strengths and weaknesses. Even though this platform provides multiple and detailed data, it does not support transfers.

Last but not least, a tool for scouts is Scisports (https://www.scisports.com/). It has three main functionalities: recruitment, performance analysis, and data delivery. Although it provides the recruitment, it does not support team building again. It only gives some extensive filters and criteria (like age ranges, nationalities, and contract end-dates).

## 3. Materials and Methods

Yogi Berra, the baseball legend, said ’*Baseball is 90% mental. The other half is physical’*. He suggested that the technical and physical aspect of a player (his agility, technique, height, etc.) can be less important, when it comes to the potential success. Thus, in this work, we decided to analyse the full overview of a football player: not only technique and his physical parameters, but also his psychological state. In order to obtain this goal, we propose using the parameters that are listed in Table 1, Table 2 and Table 3, which contain the physical, technical, and psychological elements, respectively.

The metrics that are included in the physical aspect are the statistics showing the number of games played and the duels won against the opponent. We have chosen them in order to reflect the performance and motor skills of the player.

The statistics that make up the technical aspect have been selected to reflect the contribution of the player to the game of the whole team. The key elements of soccer, such as scoring a goal, dribbling, or passing, can be presented in two ways: numerically (as an effect) or as a percentage (as an effectiveness). Soccer is a team sport, so choosing only one of these variants could create false results, e.g., if the team does not create shooting situations, even the best striker will not score a large number of goals, adequately, with a large number of situations a player with poor efficiency will score goals.

The common saying ’*statistics don’t play*’ says that taking only basic statistics into account does not always reflect the skills of the player and his contribution to the team. In order to best reflect the football profile, we have decided to also include metrics, such as big chance created or mistake leads to a goal.

The psychological aspect consists of the statistics of the cards received, contact with the ball, and the parameters that indicate the likely acclimatization of the player in the new club. Penalties received for fouls are indicative of the level of aggression of the player. The number of contacts with the ball may indicate the player’s participation in the team’s game.

### 3.1. Dataset-Data Acquisition and Pre-Processing

Because the sport statistics are considered to be very informative for fans, journalists, and the people placing bets, they are widely accessible online. The WhoScored website (https://www.whoscored.com/) provides many match statistics, while Transfermarkt (https://www.transfermarkt.com/) focuses on transfers. The other website providing numerous pieces of information is Sofascore (https://www.sofascore.com/), which publishes detailed statistics for more than 20 different sport disciplines.

We had to download a big portion of data in order to create the dataset that would be suitable for machine learning. Thus, we decided to create a Java applet that downloads and pre-processes the raw data available on the Sofascore website. The collections of our database includes statistics of nearly 4700 players from 156 clubs belonging to the eight most popular leagues (based on UEFA ranking). As mentioned, the application has two main tasks to perform. Firstly, it downloads the selected data (the parameters that are listed in Table 1, Table 2 and Table 3). Secondly, it processes the data, e.g., some parameters are available online as a number, while we found it more useful to have the number per match. Finally, the application produces the CSV file that can be used for machine learning. The generated file used for machine learning has the following data columns: average age of the target team; age of the player; compliance with the type of the target league; nationality match; value of the technical aspect of the team; value of the mental aspect of the team; value of the physical aspect of the team; value of the technical aspect of the player; value of the mental aspect of the player; value of the physical aspect of the player; and, flag indicating successful transfer.

In order to create the dataset, we gathered together the pieces of information concerning the transfers that were made in the most powerful professional football leagues. The dataset contains the transfers from: La Liga—Spain, Serie A—Italy, Bundesliga—Germany, Ligue 1—France, Premjer-Liga—Russia, Primeira Liga—Portugal, and Premiership and Championship—UK. Those leagues are considered the best worldwide and, basically, the transfers were made between the clubs representing those leagues.

**Figure 1:**
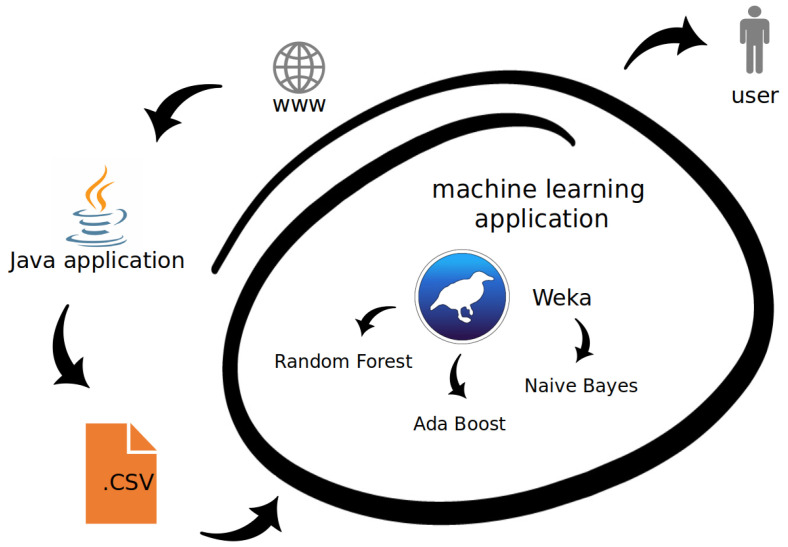
The dataflow of the proposed method: data acquisition, pre-processing, machine learning, and the result.

The dataset contains the parameters obtained from four seasons: 2019/20, 2018/19, 2017/18, and 2016/17. The values were modified by weights-the most recent season is more important than the other ones. Thus, we introduced the weights: 1.0, 0.8, 0.5, and 0.3, respectively. In a football game, there is one position with specific characteristics and stats: a goalkeeper. He neither scores nor participates in aerials and passes. Hence, the goalkeepers can influence the results of the predictions and it was decided to omit them in the dataset. In the dataset, there were 3482 records, each corresponding to a single football player transfer. The data acquisition step and all the subsequent elements of the proposed approach are presented in Figure 1.

**Figure 2:**
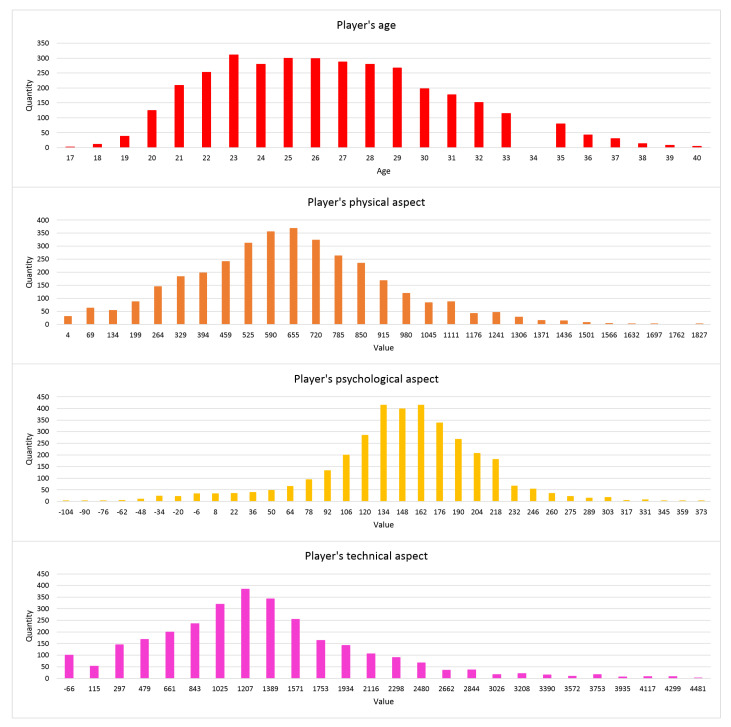
The distributions of some parameters in the datasets.

In Figure 2, the distributions of the parameters in the dataset are presented. In this figure, four bar charts are visible. They present the quantity of transfers in the dataset concerning the specific value of a parameter: age, physical aspect, psychological aspect, and technical aspect. As visible, they can be described as looking like the Gaussian distribution. In fact, they represent the real situation; most of the players are on the average level of technique, psyche, and physics, while only some players have extraordinary skills.

### 3.2. Successful Transfer Definitions

We have to define what a successful transfer really is in order to predict whether a transfer could be successful. For the purpose of this research, we have defined three different metrics of success.

Firstly, a successful transfer is the transfer affecting high results in all of the player’s aspects. Thus, we provide the total player assessment equation expressed with Equation (1), where: *t*, *p*, and *f* are the technical, psychological, and physical aspects of players; α, β, and γ are the weights of the corresponding aspects; *x* is the impact parameter, x=1, when the parameter has a positive impact on the player assessment, whereas x=−1 for the negative impact. Finally *a*, *b*, and *c* are the weights of the respective aspect, i.e., technical, psychological, and physical. If the player assessment that is calculated at the end of the season after the transfer is equal to or greater than the predefined threshold, this transfer can be considered as successful. After some consideration, we selected two types of thresholds (6.8 points and 7.2 points in 10 points scale).

(1) $$P=a\cdot \sum_{l=1}^{k}xt_{l}\alpha_{l}+b\cdot \sum_{i=1}^{n}xp_{i}\beta_{i}+c\cdot \sum_{j=1}^{m}xf_{j}\gamma_{j}$$

Secondly, the success of the transfer can rely on the overall quality of the target team, e.g., it is very difficult to score frequently, when the partners from the team are weak and do not properly support the player. Thus, in the second approach, we compare the player assessment (Equation (1)) with the average assessment of the team. If the person’s evaluation is greater than or equal to the average value, the transfer is considered to be successful.

The last successful transfer definition is based on the average team player’s assessment again, but it introduces one modification: the average is calculated taking only 18 scores (starting eleven + 7 substitutions). It is because, generally speaking, other players do not appear in the line-up.

Because of the fact of using three different football player transfer definitions, the ratio between the successful and unsuccessful transfers is not constant for all experiments. For example, it was 2024:1458 for definition 1, 2378:1104 for definition 2, and 2384:1098 for the last definition.

### 3.3. Machine Learning

The dataset was randomly divided into the training set (70%) and testing set (30%). Each experiment was repeated three times while using the three-fold cross validation in order to prove the lack of dependency on the data.

In the classification stage of the research, we used the industrial standards and state of the art ML techniques, namely the Random Forest, Naive Bayes, and AdaBoost algorithms. The Random Forest consists of a number (in this research-10) of individual decision trees that operate as an ensemble. Each individual tree in the Random Forest outputs a class prediction and the class with the most votes becomes our model’s prediction. The Naive Bayes classifier is a probabilistic machine learning model, whereas Adaboost was designed to use short decision tree models, each with a single decision point. Such short trees are often referred to as decision stumps. However, in further studies, we plan to deploy deep learning approach or artificial neural networks (as presented in [26] in order to predict the success of the football player transfer.

## 4. Results and Discussion

### 4.1. Market Value Analysis

Eden Hazard played seven successful seasons scoring 110 goals in 352 games for Chelsea. He won two Premier League titles, two Europa Leagues, the FA Cup, and League Cup. In summer 2019, he signed a €150 M contract for Real Madrid. This transfer was one of the most expensive transfers involving an English club in history (https://www.telegraph.co.uk/football/2019/06/07/eden-hazard-leaves-chelsea-real-madrid-move-worth-130m/). However, the transfer cannot be considered successful so far. Hazard suffered two injuries, which made him miss numerous matches and, in fact, the whole season for Real Madrid. The market value of Eden Hazard has decreased significantly since 2019. In June 2019, it was €150 M, while, in April 2020, it was only €80 M.

**Figure 3:**
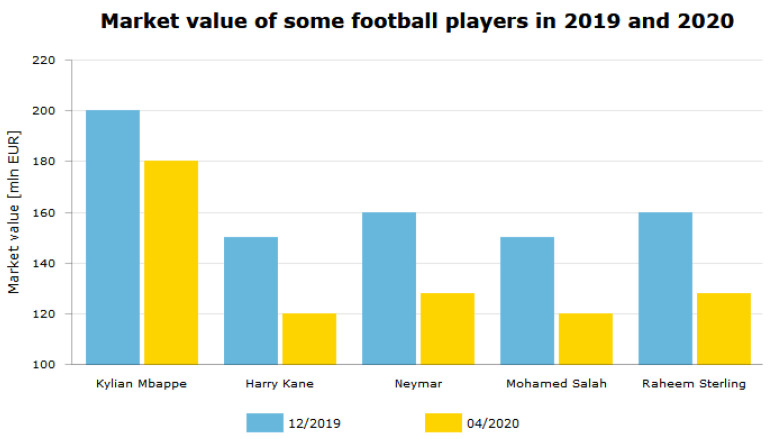
The market value (in €M) of some selected football players in 2019 and 2020: Kylian Mbappe, Harry Kane, Neymar, Mohamed Salah, and Raheem Sterling.

In Figure 3, the market value of some selected football players is presented. It could be noticed that the market value significantly decreased between December 2019 and April 2020. This downturn was caused by the COVID-19 pandemic and it can be easily visible in numerous players’ statistics.

Thus, the Eden Hazard’s case and the COVID-19-related downturn prove that the footballer’s market value and the transfer value are not always reliable and, thus, in our analysis, we decided to skip all of the data concerning money.

### 4.2. Obtained Results

We decided to present the results that were obtained from various experiments. They differ between each other in different successful transfer definitions, which were described in detail in Section 3.2: R6.8-definition 1, threshold = 6.8, R7.2-definition 1, threshold = 7.2, A1.0-definition 2, A0.8-definition 2, the target team average multiplied by factor 0.8, S1.0-definition 3, and finally, S0.8-definition 2, the target team average multiplied by factor 0.8. The results are expressed using four measures: accuracy, precision, recall, and F1-score. The definitions of the measures are expressed with Equations (2)–(5), where TP-true positives, FP-false positives, TN-true negatives, and FN-false negatives. Table 4 presents the results.

(2) $$Acc=\frac{TP+TN}{TP+TN+FP+FN}$$

(3) $$precision=\frac{TP}{TP+FP}$$

(4) $$recall=\frac{TP}{TP+FN}$$

(5) $$F1-score=2\cdot \frac{precision\cdot recall}{precision+recall}$$

After executing the first part of the experiments, we decided to choose the most promising definition of a successful transfer and weights variant, namely the variant A and the definition A0.8 (the values in bold from the Table 4). Subsequently, the parameters *a*, *b*, and *c* from Equation (1) were modified. They define the importance of the technical, physical, and psychological aspects of each player. Table 5 presents the obtained results from this experiment. Some experiments were also performed with the Naive Bayes and AdaBoost as a classifier in order to present the comparison of the Random Forest with the other ML methods. Table 6 presents the results of this comparison.

## 5. Threats to Validity

Arsene Wenger, the veteran Arsenal boss, admitted that football, as well as any other sport, is mainly unpredictable https://www.irishexaminer.com/sport/soccer/arid-20321805.html. It may lead to the assumption that the proposed approach can just be a suggestion. The transfer supporting may contain a flaw and the predictions that are made by the proposed method have to be treated more as suggestions, since there are always unpredictable factors (e.g., family life/problems, serious injuries).

Another threat to validity is the situation that can be currently observed. The 2020 season was interrupted and the training cycle was destroyed in most leagues all over the world due to the global COVID-19 pandemic. It may cause some misleading data in statistics and, consequently, it may lead to failed predictions in the future.

Last but not least, the differences between leagues may be another threat to validity. It is very difficult to compare the leagues from different countries. The proposed method was tested against the data regarding the players and transfers from the best leagues.

Additionally, our data from the top leagues only concerned the male players/leagues. It is the future plan to evaluate whether the same approach will work well on the data regarding female football players.

As for construct validity, the used dataset is believed to be representative for the top football leagues, and it is well constructed based on the open and available data. As for external validity, we are aware that the proposed method is based on top leagues and that it can be biased and provide less promising results for weaker players and teams.

## 6. Conclusions

In this paper, we present the possible approach to the professional team building by means of the machine learning based tool. The proposed solution provides promising results and it can support a scout or a team manager in the process of transfer planning. Nevertheless, as mentioned in the Section 5, there are some threats to validity that can affect the obtained results and the whole approach. Additionally, as mentioned in [27], sport is unpredictable. Hence, the indications coming from ML should be treated solely as the pieces of advice.

In this research, unlike some other approaches that have been recently published (e.g., [28]), we only used the realistic data. We obtained the dataset from the online sources and hope that it can represent the real situation in football.

The obtained results also depend on the successful transfer definition and the weights of the selected parameters. In this paper, we provide three various definitions and three different weights for each parameter. It is also remarkable that we do not only take the technical and physical parameters of a professional football player into account, but also his psychological state.

In the near future, we are going to further improve the presented solution. The possible extension would include adding more classifiers (as a neural network) and building ensembles. Apart from that, we are going to verify the solution on other data, possibly coming from a greater number of (less popular) leagues. Last but not least, a possible extension is an attempt to move the proposed approach to other ball-sports, e.g., volleyball or basketball, which would definitely need the parameters’ lists to be redefined.

## Figures and Tables

**Table 1:** Players’ physical parameters used in the football team building and transfer supporting method.

Parameter Name	Impact	Weight A	Weight B	Weight C
Matches played	positive	2.0	2.0	3.0
Matches played from the beginning	positive	1.0	1.25	2.0
Aerials won	positive	1.5	1.5	2.0
One to one on the ground won	positive	2.0	2.0	2.0

**Table 2:** Players’ technical parameters used in the football team building and transfer supporting method.

Parameter Name	Impact	Weight A	Weight B	Weight C
Goals from the penalty box	positive	30.0	50.0	10.0
Goals out of the penalty box	positive	50.0	50.0	20.0
Goals with right leg	positive	20.0	25.0	0.0
Goals with left leg	positive	20.0	25.0	0.0
Goals with head	positive	20.0	25.0	0.0
Goals from the penalty kick	positive	20.0	25.0	5.0
Participation in team goals	positive	100.0	120.0	20.0
Shoots per match	positive	20.0	30.0	2.0
Penalty kick obtained	positive	10.0	10.0	3.0
Successful dribbles per game	positive	1.0	1.25	5.0
Accurate passes per game	positive	1.0	1.25	5.0
Successful crosses and corners	positive	1.0	1.25	5.0
Assists	positive	30.0	50.0	20.0
Key passes per game	positive	20.0	25.0	5.0
Big chance created	positive	10.0	30.0	5.0
Successful long passes	positive	1.0	1.25	5.0
Successful passes in own half	positive	1.0	1.25	5.0
Successful passes in opposition half	positive	1.0	1.25	5.0
Tackles per game	positive	25.0	25.0	10.0
Interceptions per game	positive	100.0	100.0	10.0
Was fouled per game	positive	50.0	50.0	3.0
Big chance missed	negative	10.0	10.0	2.0
Challenges lost per game	negative	20.0	20.0	2.0
Mistake leads to a shot	negative	30.0	30.0	5.0
Mistake leads to a goal	negative	30.0	30.0	5.0
Fouls per game	negative	30.0	30.0	5.0
Provoked penalties	negative	5.0	5.0	5.0
Offsides	negative	20.0	20.0	2.0
Possession lost	negative	5.0	5.0	1.0

**Table 3:** Players’ psychological parameters used in the football team building and transfer supporting method.

Parameter Name	Impact	Weight A	Weight B	Weight C
Age (if it is within the desired range)	positive	0.3	0.3	0.1
Nationality (if in compliance with team members)	positive	0.8	50.0	10.0
League characteristics (if in compliance)	positive	0.7	50.0	10.0
Ball touches per game	positive	1.0	1.25	3.0
Yellow cards	negative	5.0	5.0	2.0
Red cards directly	negative	5.0	5.0	3.0
Red cards indirectly (second yellow)	negative	5.0	10.0	5.0

**Table 4:** The results obtained from experiments for different successful transfer definitions.

Variant	Measure	R6.8	R7.2	A1.0	A0.8	S1.0	S0.8
A	accuracy	0.6676	0.5974	0.6909	**0.8108**	0.6903	0.8153
	precision	0.6463	0.5857	0.6897	**0.8467**	0.7217	0.8170
	recall	0.6677	0.5977	0.6910	**0.8107**	0.6903	0.8153
	F1-score	0.6562	0.5916	0.6902	**0.8283**	0.7048	0.8162
B	accuracy	0.6632	0.6089	0.6632	0.7907	0.6641	0.8022
	precision	0.6823	0.5600	0.7257	0.7800	0.7067	0.8007
	recall	0.6633	0.6087	0.6633	0.7907	0.6640	0.8023
	F1-score	0.6697	0.5833	0.6922	0.7852	0.6837	0.8015
C	accuracy	0.6612	0.6041	0.7231	0.8258	0.7174	0.8360
	precision	0.6750	0.6072	0.5237	0.5757	0.5363	0.5593
	recall	0.6613	0.6043	0.7230	0.8260	0.7173	0.8360
	F1-score	0.6672	0.6058	0.5094	0.5665	0.6138	0.6702

**Table 5:** The results obtained from experiments for different weights of aspects (a—technical, b—psychological, and c—physical).

Parameters	Accuracy	Precision	Recall	F1-Score
a = 1.0, b = 1.0, c = 1.0	0.8108	0.8467	0.8107	0.8283
**a = 0.8, b = 1.2, c = 1.0**	**0.8246**	**0.8357**	**0.8247**	**0.8300**
a = 1.2, b = 0.8, c = 1.0	0.8080	0.8210	0.8077	0.8141
a = 0.9, b = 1.2, c = 0.9	0.8121	0.8193	0.8120	0.8154

**Table 6:** Results that were obtained from experiments for different classifiers.

Classifier	Accuracy	Precision	Recall	F1-Score
Random Forest	**0.8246**	**0.8357**	**0.8247**	**0.8300**
Naive Bayes	0.5627	0.8240	0.5627	0.6261
AdaBoost	0.8274	0.7970	0.8273	0.8119
